# Distinct Types of White Matter Changes Are Observed after Anterior Temporal Lobectomy in Epilepsy

**DOI:** 10.1371/journal.pone.0104211

**Published:** 2014-08-04

**Authors:** Dorian Pustina, Gaelle Doucet, James Evans, Ashwini Sharan, Michael Sperling, Christopher Skidmore, Joseph Tracy

**Affiliations:** 1 Thomas Jefferson University, Department of Neurology, Philadelphia, Pennsylvania, United States of America; 2 Thomas Jefferson University, Department of Neurosurgery, Philadelphia, Pennsylvania, United States of America; Centre Hospitalier Universitaire Vaudois Lausanne - CHUV, UNIL, Switzerland

## Abstract

Anterior temporal lobectomy (ATL) is commonly adopted to control medically intractable temporal lobe epilepsy (TLE). Depending on the side of resection, the degree to which Wallerian degeneration and adaptive plasticity occur after ATL has important implications for understanding cognitive and clinical outcome. We obtained diffusion tensor imaging from 24 TLE patients (12 left) before and after surgery, and 12 matched controls at comparable time intervals. Voxel-based analyses were performed on fractional anisotropy (FA) before and after surgery. Areas with postoperative FA increase were further investigated to distinguish between genuine plasticity and processes related to the degeneration of crossing fibers. Before surgery, both patient groups showed bilateral reduced FA in numerous tracts, but left TLE patients showed more extensive effects, including language tracts in the contralateral hemisphere (superior longitudinal fasciculus and uncinate). After surgery, FA decreased ipsilaterally in both ATL groups, affecting the fornix, uncinate, stria terminalis, and corpus callosum. FA increased ipsilaterally along the superior corona radiata in both left and right ATL groups, exceeding normal FA values. In these clusters, the mode of anisotropy increased as well, confirming fiber degeneration in an area with crossing fibers. In left ATL patients, pre-existing low FA values in right superior longitudinal and uncinate fasciculi normalized after surgery, while MO values did not change. Preoperative verbal fluency correlated with FA values in all areas that later increased FA in left TLE patients, but postoperative verbal fluency correlated only with FA of the right superior longitudinal fasciculus. Our results demonstrate that genuine reorganization occurs in non-dominant language tracts after dominant hemisphere resection, a process that may help implement the inter-hemispheric shift of language activation found in fMRI studies. The results indicate that left TLE patients, despite showing more initial white matter damage, have the potential for greater adaptive changes postoperatively than right TLE patients.

## Introduction

Temporal lobe epilepsy (TLE) is the most frequent type of epilepsy, with its intractable form commonly leading to surgical treatment [1]. White matter disruptions have been reported in TLE patients before undergoing any surgery. These disruptions involve tracts both proximal to the epileptogenic area, such as, the uncinate, the parahippocampal, and the inferior fronto-longitudinal fasciculi [2]–[10], as well as distal tracts, such as, the arcuate, the fornix, the cingulate, the corpus callosum, etc. [2], [3], [6]–[8], [10]–[13]. In an attempt to identify factors that contribute to WM abnormalities and the potential for reorganization, specific distinctions have been made between clinical features, i.e. left vs. right TLE patients, with vs. without mesial temporal sclerosis (MTS), and early vs. late seizure onset. Typically, more widespread WM disruptions are found in left TLE patients, MTS patients, and those with early onset seizures [3], [4], [7], [8], [14], [15].

The distinction between left and right TLE is particularly important given that the left hemisphere is dominant for language processes in the vast majority of cases. Before surgery, normal asymmetries are altered depending on the side of the pathology. For instance, the normal leftward asymmetry of the arcuate fasciculus [16], [17] is disturbed in left, but not right, TLE [4], [15], [18]. In contrast, the uncinate fasciculus has a normal rightward asymmetry with higher FA and more fibers on the right [19], [20], creating the possibility of higher WM risk in those tracts specifically bearing the burden of seizure dispersion (i.e., the uncinate [21]).

Resective surgery further modifies white matter tracts, in part because of direct resection of certain tracts during anterior temporal lobectomy (ATL), and in part because of the cessation of the seizure burden in about 70% of patients [22], [23]. The distinction between degradation processes (i.e. Wallerian degeneration) and behaviorally adaptive changes is crucial for understanding the substrates of neurocognitive plasticity. Yet, while many studies report WM degeneration after surgery [24]–[29], few studies have reported the opposite effect, that is, improvement of WM diffusion properties [25], [27]. These reports indicate potentially neuroplastic changes exclusively in left ATL patients, only ipsilateral to the resection, while right ATL were either absent from the study [25] or showed minimal potential for neuroplasticity [27]. A correlation of ipsilateral WM reorganization and verbal fluency scores have been reported by Yogarajah et al. [27], leading the authors to conclude that language processes rely on intra-hemispheric plasticity. The study, however, lacked a control group, and, thus, was unable to determine whether WM status was normal before or normalized after surgery. In addition, postoperative measurements were limited to a few months post-surgery [27], an amount of time insufficient to capture changes that continue for at least one year post-surgery [25], [30]. A recent study by Winston et al. [30] provided additional evidence that both left and right ATL patients show FA increases ipsilateral to the resection in both ATL groups postoperatively, running counter to the account proposed by Yogarajah et al. [27] regarding functional adaptations in language involving solely ipsilateral (dominant hemisphere) changes following left ATL.

The aim of the present study is to establish whether left and right TLE patients (LTLE and RTLE; called LATL and RATL after surgery) display different potential for long-term WM reorganization, and to distinguish areas showing FA increase caused by crossing fiber degeneration from those showing genuine neuroplasticity. We conduct whole brain analyses, but in a second stage of analyses focus on tracts the literature suggests are associated with language functioning. To achieve this goal we use measures of diffusion tensor imaging (DTI) obtained from patients and matched controls at comparable time intervals. Based on previous findings [25], [27], there is reason to suspect that post-surgical white matter plasticity would most likely occur in the corona radiata.

## Materials and Methods

### Participants

A total of 24 patients with refractory, unilateral and focal temporal lobe epilepsy were recruited from the Thomas Jefferson University Comprehensive Epilepsy Center (12 left TLE; 12 right TLE). All patients had solely unilateral temporal pathology, and all were recommended for anterior temporal lobe resection as treatment for their intractable TLE. Details of the algorithm for surgical decision making are described in Sperling et al. [31]. A combination of EEG (at least 96 hours), video recording, MRI, PET, and neuropsychological testing was used to localize the seizure focus. Expert board-certified neuroradiologists and epileptologists, by consensus decision, classified patient neuropathology and seizure type. When the data were not convergent between the various tests, intracranial electrodes were implanted to determine the seizure focus through electrocorticography (three of 24 patients).

Participants were excluded from the study for any medical illness with central nervous system impact other than epilepsy; prior or current alcohol or illicit drug abuse; extratemporal epilepsy; present or past neoplasia; psychiatric diagnosis for any Axis I disorder listed in the Diagnostic and Statistical Manual of Mental Disorders – IV. Depressive Disorders were allowed in the patient sample, given the high co-morbidity of depression and epilepsy [32]. Patients with mental retardation (Full-Scale IQ <70) who were likely to be unable to cooperate with the MRI examination were also excluded. Table S1 displays relevant demographic and clinical characteristics of the subjects.

Another group of 12 healthy normal subjects were recruited, matching the TLE patients in age and handedness. A health screening measure checked and avoided cases of neuropathology, learning disorder, head trauma, drug abuse, alcohol abuse, seizures, or psychiatric disorders.

### Ethics statement

Participants provided written informed consent, and the study was approved by the Institutional Review Board for Research with Human Subjects at the Thomas Jefferson University.

### MRI acquisition

Images were acquired in a Philips Achieva 3T scanner (Amsterdam, the Netherlands) using an 8-channel SENSE head coil. No single group was scanned on a schedule or time different than the other groups, thereby avoiding bias related to temporally dependent scanner calibration.

A single-shot spin-echo EPI pulse sequence (TE = 90 ms, TR = 8609 ms, FOV = 230 mm, 66 slices, 2 mm thickeness, 0 mm gap, 128×128 acquisition & reconstruction matrix, 1.8 mm×1.8 mm×2 mm voxel size) was used to acquire in axial plane 32 non-collinear diffusion volumes (b-factor = 850 s/mm^2^), and three non-diffusion volumes (b-factor = 0 s/mm^2^). Fat suppression was achieved using a standard SPIR (spectral pre-saturation with inversion recovery) technique. The sequence was repeated three times for each subject to increase signal to noise ratio.

A T1-weighted anatomical MP-RAGE volume was collected during the same session in sagittal orientation with in-plane resolution of 256×240 and 1 mm slice thickness (isotropic voxels of 1 mm^3^; TR = 650 ms, TE = 3.2 ms, FOV 256 mm, flip angle 8°).

### DTI Pre-Processing

Images were imported into DTIstudio software [33] from the Philips PAR/REC format. Subject motion and eddy currents were corrected in a single step by registering diffusion weighted (DW) volumes on the averaged non-DW (B0) volume. A mutual information affine algorithm was used for this purpose. All our subjects had originally 3 DTI repetitions. We removed a single dataset from two left TLE, two right TLE, and a control, because of data corruption or excessive head motion. Tensors were calculated with a standard linear regression in DTIstudio. Maps of DTI measurements, such as, fractional anisotropy (FA), parallel diffusivity (λ∥), perpendicular diffusivity (λT), and mode of anisotropy (MO), were created based on the six unique tensor matrix elements. Parallel and perpendicular diffusivities provide complementary information on underlying biological processes causing FA change, and have been previously associated with axonal and myelin integrity, respectively [34]–[36]. MO was calculated in Matlab (The Mathworks Inc., USA) using the algorithm published by Ennis and Kindlmann [37]. This measure is mathematically independent from FA, and characterizes the shape of the tensor from linear to planar based on differences between the three lambda values. Some have argued that MO can be used as an index of crossing fibers [37]–[39].

Inter-session registration was performed in SPM software (SPM8; Wellcome Trust Centre for Neuroimaging, UCL, UK) using post-surgery FA (FApost) as source and pre-surgery FA (FApre) as target. This step used the normalization option in SPM8 to perform an affine transformation followed by finer non-linear transformations (2 mm smoothing of source and target during calculation, 16 non-linear iterations, 25 mm cutoff frequency). Both source and target volumes were skull stripped and the resection area was masked out for ATL patients. The resection mask was created by manually drawing the patients' resected area on the their post-surgical T1 image previously coregistered to FA. MRIcron software was used for this purpose [40]. The result from this longitudinal registration step was visually inspected for each subject and no cases of poor registration were observed. Joint histograms were created to further examine the quality of the registration. These histograms are presented in Supplementary Material (File S1) for all the participants. The longitudinal registration transformation was applied to the other post-surgery maps (λ∥, λT, MO) resulting in coregistered pre- and post-surgery images.

Normalization in MNI space was performed in Diffeomap [33] for all maps at the same time (both pre and post). The transformation matrix was calculated from the pre-surgical maps in a two-step procedure consisting of an affine transformation followed by a single channel LDDMM (large deformation diffeomorphic metric mapping; [41]). The single subject template provided with Diffeomap was used as final target. The affine transformation was driven by respective B0 images of the subject and the template, while LDDMM was performed on FA images using a matching threshold of p<0.005. To improve the results, skull-stripped versions of B0 and FA maps were used for both above steps. The matrix obtained after the two normalization steps was applied to pre- and post- maps at the same time. Finally, the resulting maps (181×217×181 voxels, 1 mm^3^ isotropic) were smoothed with a 4 mm full width half maximum Gaussian filter to increase signal-to-noise ratio, improve eventual misregistrations, and increase the normality of the distribution. The smoothing kernel size was decided based on previous evidence showing a decrease in sensitivity for larger smoothing kernels [20].

### Statistical analyses

Whole-brain statistical analyses were performed on FA maps using SPM8 software. Pre-surgery status and post-surgery changes in FA were analyzed with separate ANOVAs. Each ANOVA contained a three-level independent factor (i.e., the two patient groups and the control group). The key contrasts tested for differences in FA between the two patient groups (t-tests), and between the patient groups and controls (t-tests). As our goal was to be sensitive to any tract showing plasticity effects in whole brain analyses, we utilized a threshold of p<0.001 (uncorrected), a t-value greater than 3, and an extent threshold of k = 65. The cluster threshold was above the expected voxels per cluster estimated estimated by our ANOVAs and allowed us to achieve an adequate spatial resolution for each cluster in acquisition space, corresponding to 10 voxels within the scanning resolution of 1.8×1.8×2 mm. We followed up whole brain analyses with cluster-specific analyses on tracts showing potential plasticity. More rigorous FWE corrected findings are described as well. To analyze pre- to post-surgery FA changes, a difference image (Δ delta) was created for each participant. This image was obtained by subtracting pre and post images (FApre – FApost). Proper coding of the contrast values allowed to test for FA decreases (FApre minus FApost) and increases (FApost minus FApre) across the scanning sessions. An inclusive masking procedure was used to allow only voxels where patients showed change to be part of the comparison with patients. For example, to obtain FA decrease in patients, only voxels that reached a t value >2.73 (p<.005) for FA decrease in the patient group (FApre minus FApost) were part of the contrast with the control group, using an inclusive mask.

Significant whole-brain results were assigned to specific white matter tracts by overlaying thresholded t-statistic maps on the single subject MNI template used during normalization. This template is parcellated in 118 different white and gray matter structures (brain parcellation map, BPM type III; [42]). The number of significant voxels on each tract, along with the percentage of the tract affected, is displayed in Tables S2, S3, S4. Average t-values, p-values, and effect sizes were computed for the voxels affecting each tract. Gray matter structures (except hippocampus) and tracts composed of less than 10 voxels were excluded from the report.

Clusters of interest were further investigated using the other DTI measures (λ∥, λT, MO). The stability of results was checked by computing z-scores for patients with respect to control subjects. Moreover, reliability analyses in controls, involving intra-class correlations, were run to verify that any change observed in patients was gauged against stable findings in controls. Follow-up analyses and group analyses on demographic measures (i.e., age, IQ, scan intervals, etc.) were performed in SPSS software (IBM SPSS Statistics for Windows v.20, Armonk, NY).

### Calculation of FA Laterality, Hemispheric Dominance, and Cerebral/Resection Volumes

FA asymmetries were computed for two language tracts, the superior longitudinal fasciculus (SLF) and the uncinate (UNC). To compute the laterality index (LI), FA values were averaged in SLF or UNC parcels of the parcellation map. The following LI formula was used: (left - right)/(left + right).

Regarding hemispheric dominance for language, we derived a binary categorization from available task-based fMRI or a reliable measure of handedness. fMRI-based dominance was determined with a verb generation task, requiring covert generation of a verb in response to a viewed noun [43]. Using a bootstrap algorithm [44], laterality was computed from fMRI maps and subjects were categorized left hemispheric (−1<LI<−0.4), right hemispheric (0.4<LI<1), or bilaterally dominant (−0.4<LI<0.4). A total of eight out of 12 LTLE, and nine out of 12 RTLE patients had available fMRI data, with all, except one RTLE patient, showing left hemispheric dominance. The one RTLE patient had right hemispheric dominance. The remaining patients were categorized using the Edinburgh Handedness Scale (left hemispheric if score >70) [45], and all were right-handed and considered likely to be left hemispheric dominant. In sum, all 12 LTLE patients were left hemisphere dominant (100%), and 11 of 12 right TLE patients were left hemisphere dominant (92%). Control subjects were all left hemispheric dominant confirmed with fMRI.

To make sure that the two patient groups were comparable in resection and brain volume, we calculated the volume of resection and total cerebral volume. These values were obtained by registering and segmenting pre- and post-surgery T1 images. A difference image was created by subtracting probability maps of gray and white matter pre-to-post surgery. The resulting image contained a clear view of the missing cerebral tissue, which was then drawn in MRIcron. The resection volume was computed by the number of voxels in this mask. In parallel, total cerebral volume was obtained from the pre-surgery segmented probability maps by counting voxels with p>0.1 in gray/white matter. The ratio between the resection volume (numerator) and the total cerebral volume (denominator) gave the resection ratio.

### Neuropsychology scores

Phonemic and semantic fluency scores were obtained from patients before and after surgery as part of a standard battery of neuropsychology tests. The phonemic fluency score consisted of the total number of words produced in 60 seconds starting with a specific letter, summed over three distinct trials (e.g., F, A, and S; Controlled Oral Word Association Test; [46]). The semantic fluency score consisted of the total number of animal names produced in 60 seconds (Animal Naming; [46]).

## Results

The two TLE groups did not differ in age, IQ, epilepsy duration, cerebral volume, and resection ratio (all p>0.1). The resection size, although not statistically different, showed a trend to be bigger in the right hemisphere (T(22) = −1.8, p = 0.09). A significant difference was found in the post-surgery time interval, for which LTLE were found to have waited longer between surgery and the second scan (T(22) = 2.18, p<0.05; LTLE = 1.7 y, RTLE = 0.79 y). Closer data inspection exposed an outlier in the LATL group (3.4 STDs from the group average). Removal of this case dropped the significance to p = 0.09, leaving only a trend (T(12.9) = 1.87, corrected for variance inhomogeneity). Given the sensitivity of this variable for the interpretation of the results, an additional ANOVA on whole-brain post-surgery changes (ΔFA) was run with this measure as covariate. Another critical clinical measure, duration of epilepsy, was used as covariate in pre-operative comparisons in a separate ANOVA. Regarding mesial temporal sclerosis (MTS), six left and three right TLE patients were found to have ipsilateral MTS (Pearson Chi-Square = 1.6, p = 0.2; no patient had bilateral MTS). Regarding gender distribution, the left TLE group had a male/female ratio of 2/10 while the right TLE group had a ratio of 7/5 (Pearson Chi-Square = 4.4, p = 0.04). Male/female ratio in healthy controls was 9/3.

Detailed information about the subjects participating in the study is presented in Table S1, including the anti-epileptic drugs patients took before and after surgery.

### FA Status in TLE Groups Prior to Surgery (FApre)

A direct comparison of TLE groups showed no significant results. The following results refer to the comparison of TLE groups with the control group.

Compared to controls, both TLE groups showed reduced FA and no indication of increased FA. The LTLE patients showed more spatially extensive reduced FA (∼40,000 voxels), affecting most of the tracts bilaterally, while RTLE showed a more limited pattern of reduced FA (∼7,500 voxels), affecting mainly unilateral tracts (Table S2).

Regarding the uncinate (UNC), a tract that connects the orbitofrontal cortex with the anterior temporal lobe, we found only contralateral reduced FA in LTLE patients. No effect on UNC was seen in RTLE patients.

**Figure 1:**
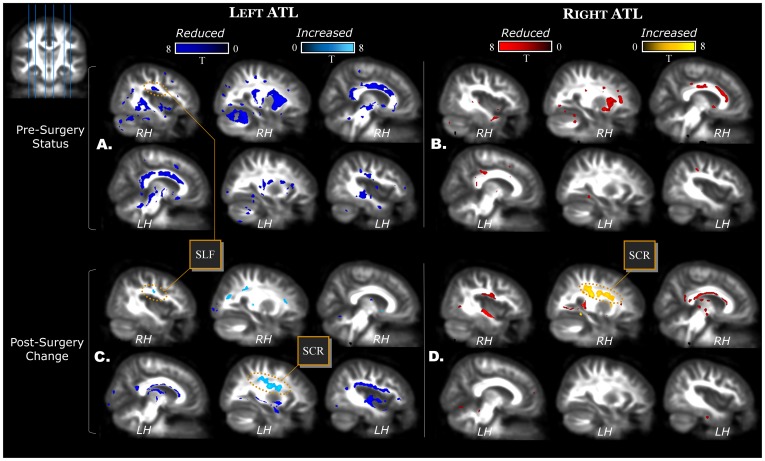
FA status in TLE patients before surgery. Left TLE in the left (panel A) and right TLE in the right (panel B). Post-surgery changes are displayed for left (panel C) and right (panel D) ATL patients. Slice positions are displayed in the upper left corner. The six slices displayed in each panel are located in MNI space x = [−40 −27 −8 +8 +28 +40]. Abbreviations indicate SLF = superior longitudinal fasciculus, and SCR = superior corona radiata.

The superior longitudinal fasciculus (SLF) contains fibers connecting the frontal lobes with the ipsilateral temporal and parietal lobes. Importantly, the arcuate fasciculus, an important connectivity pathway for language functions, is part of the SLF. The SLF showed bilateral lower FA in LTLE patients compared to controls, symmetrical in nature (10% left and 11% right hemisphere; Fig. 1), with no such reduction evident in the RTLE group.

All the limbic pathways (stria terminalis of the fornix, parahippocampal fasciculus, hippocampus, and cingulate fasciculus) showed lower FA bilaterally in LTLE patients compared to controls. RTLE patients showed bilateral lower FA only in the cingulate, while hippocampus and parahippocampal pathways were affected only in the contralateral hemisphere.

The results regarding the other WM tracts in patients compared to controls are as follows. The corpus callosum showed diminished FA in both TLE groups, but the LTLE group displayed a larger spatial extent with bilateral FA abnormalities. The inferior fronto-occipital fasciculus was asymmetrically affected more in the right hemisphere in both patient groups, independent of the side of TLE. Similarly, the internal/external capsules, and the corona radiata showed more extensive reduced FA in the right hemisphere in both TLE groups. An area covering the right anterior corona radiata and right internal capsule showed reduced FA in both TLE groups and the highest statistical effect size (Fig. 1, x = −27; see ALIC in Table S2). This was the only area that remained significant after FWE correction. Non-specific white matter in the frontal, temporal, and parietal lobes, which was not assigned to any major known tracts, showed reduced FA in both patient groups. However, the LTLE group had extensive bilateral FA reduction, while the RTLE group had fewer voxels affecting non-specific WM.

### Pre-surgery asymmetries in the SLF and UNC

Healthy control subjects showed the normative right lateralization in the UNC (LI 95% confidence interval = −0.04, −0.12; T(11) = −4.68, p = 0.001), but did not show the expected left lateralization in SLF (95% confidence interval = 0, 0; T(11) = 0.00, p = 0.9). Compared to healthy subjects, LTLE patients showed less lateralization in the UNC (T(22) = −3.43, p = 0.002), while RTLE did not differ from controls (T(22) = 1.34, p = 0.19). Regarding the SLF, all three groups showed similar symmetric FA in this tract (mean LI: LTLE = 0.007; RTLE = 0.014; NC = 0.000; F(2,33) = 1.63, p = 0.21).

### The role of epilepsy duration in FApre

The inclusion of epilepsy duration in the pre-surgery whole-brain analysis did not alter the pattern of reduced FA observed in patient groups, with only slight changes in the number of significant voxels. The contrast on the covariate itself showed an inverse relation with FA values along prefrontal WM, corresponding to the anterior corona radiata in both patient groups. In these areas, longer epilepsy duration correlated with lower FA values. In RTLE patients, this inverse correlation was present also in the body and column of fornix.

### FA Change in healthy controls and TLE Groups After Surgery (ΔFA)

#### FA change in normal controls

One sample t-tests on the ΔFAs in control subjects revealed small clusters of FA increase or decrease. For instance, FA increase was observed in the left parahippocampal fasciculus (37 voxels) and the splenium (37 voxels). FA decrease was observed in the right posterior thalamic radiation (135 voxels), right posterior corona radiata (23 voxels), right superior longitudinal (68 voxels), and right retrolenticular part of internal capsule (45 voxels).

#### FA change in ATL groups compared to controls

**Figure 2:**
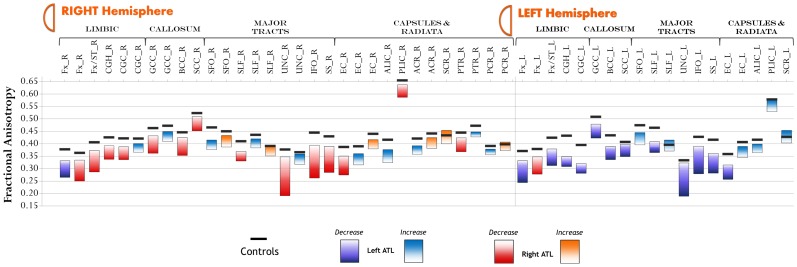
Amount of FA change for each tract pre-to-post surgery. Black lines represent average values in healthy controls. Abbreviations: Fx = body of fornix; Fx/ST = fornix (stra terminalis); CGH = cingulate of hippocampus (parahippocampal); CGC = cingulate of cingulate gyrus; GCC = genu of corpus callosum; BCC = body of corpus callosum; SCC = splenium of corpus callosum; SFO-superior fronto-occipital fasciculus; SLF = superior longitudinal fasciculus; UNC = uncinate fasciculus; IFO = inferior fronto-occipital fasciculus; SS = sagittal stratum; EC = external capsule; ALIC = anterior limb of internal capsule; PLIC = posterior limb of internal capsule; ACR = anterior corona radiata; SCR = superior corona radiata; PTR = posterior thalamic radiation.

Fig. 2 displays raw FA change pre-to-post surgery in each tract. Compared to controls, patients showed both FA decreases and FA increases.

Table S3 displays FA decreases post-surgery in LATL and RATL patients. Given the obvious effects found in the resected temporal lobe, only results outside the temporal lobe are reported. In general, both patient groups showed FA decreases ipsilateral to the resection. Only one tract, the body and column of fornix, bilaterally decreased FA in both LATL and RATL patients (y = 0 in Fig. 1). However, given the size, the mid-sagittal location, and the smoothing, a distinction between left and right fornix could not be made with certainty. All the remaining tracts showed FA decrease ipsilateral to the resection. These include the limbic pathways (stria terminalis of the fornix and parahippocampal fasciculus), the corpus callosum (body, genu and splenium), the UNC, the SLF, the inferior fronto-occipital, and non-specific WM in frontal and parietal lobes. Regarding the UNC, this tract is frequently resected in ATL surgery. Not surprisingly, it was largely affected ipsilateral to the side of surgery in LATL and RATL (97% and 99% of the tract affected, respectively). Regarding the SLF, ipsilateral FA decreases were observed in the LATL and RATL patients (12% and 2% of the tract affected, respectively). Regarding non-specific frontal and parietal WM, more extensive FA decrease was observed in LATL than RATL patients (9% and 7% in ipsilateral parietal and frontal lobes, respectively, after LATL, compared to 2% and 3%, after RATL). FWE correction restricted the results only to the uncinate (ipsilateral to the resection) and the body and column of fornix.

Though FA increases were limited compared to decreases, LATL patients showed more extensive and bilateral increases than RATL patients, including voxels in the contralateral UNC and SLF. Detailed information on FA increases is shown in Table S4. Specifically, FA increased on average by 9.3% in the contralateral SLF of LATL patients (y = −40 in Fig. 1), mainly due to a 5.7% decrease in λT, while λ∥ remained relatively stable (−0.5%). Similarly, FA increased by 12.8% in contralateral UNC of LATL patients, mainly due to a 6% decrease in λT, while λ∥ showed limited change (+0.7%). Compared to controls, eight LATL patients showed FA increase beyond 2 z-scores in contralateral SLF and seven LATL patients showed FA increase beyond 2 z-scores in contralateral UNC. Reliability analysis of FA values in the control group showed high intra-class correlation in both SLF (ICC = 0.98, p<0.001) and UNC (ICC = 0.88, p = 0.001) clusters, suggesting FA stability in these tracts over time.

**Figure 3:**
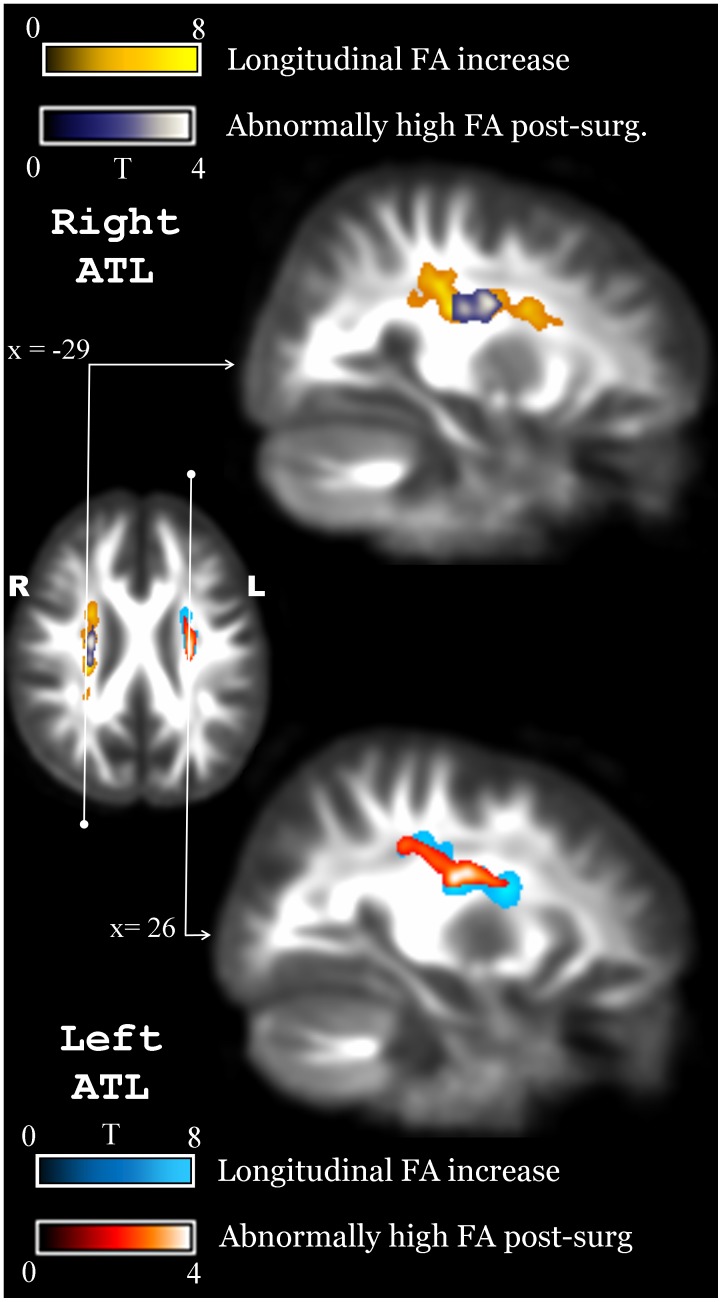
Overlap of longitudinal FA increase and cross-sectional post-surgery comparison with controls. A similar area in the ipsilateral SCR is observed to increase FA in LATL and RATL patients reaching values beyond those found in normal controls.

Notably, besides the increases in contralateral SLF and UNC in LATL patients, both ATL groups showed FA increase in large clusters ipsilateral to the resection (Fig. 3). The major tract affected by these clusters was the superior corona radiata (SCR) in both groups (in LATL, 71% of the cluster was left SCR; in RATL 67% of the cluster was right SCR). Part of the cluster involved the adjacent SLF in both groups (5% of cluster in LATL, 5% in RATL). Note, given the prominent involvement of the SCR, these prominent ipsilateral clusters will be referred to as the SCR clusters. On average, FA increased by 12% in the left SCR cluster for LATL patients and by 12.33% in the right SCR cluster for RATL patients. This increase, for LATL patients, was due to a combined effect of 5% increase in λ∥ and 4% decrease in λT. In RATL patients, FA increase was due to a combined effect of 6.3% increase in λ∥ and 3.1% decrease in λT. Compared to controls, FA increase in left and right SCR clusters was beyond 2 z-scores in 11 LATL and 10 RATL patients, respectively. Reliability analysis on the control group showed high intra-class correlation in both clusters (left SCR: ICC = 0.96, p<0.001; right SCR: ICC = 0.96, p<0.001). After applying FWE correction on FA increases, only voxels along the corona radiata (ipsilateral to resection) remained significant in both left and right ATL patients, confirming the remarkable changes in this area.

To determine if the FA increases reflect a true neuroplastic response rather than an effect caused by degeneration of crossing fibers, we investigated further four clusters of interest: the two ipsilateral SCR clusters in the respective LATL and RATL groups, and the contralateral SLF and UNC in the LATL group.

### Evidence for degeneration of crossing fibers in ipsilateral SCR

In the SCR clusters, we considered the possibility that FA may increase due to Wallerian degeneration, a plausible alternative given current DTI methodology. In areas with crossing fibers such as SCR [47], axonal degeneration in a sub-population of fibers could leave a more homogenous population, leading to an increase in FA values. If this was the case, FA values of controls would be lower than FA values of patients after surgery. We checked this possibility by running a post-operative cross-sectional whole-brain FA comparison of patients vs. controls. The standard statistical threshold (p<0.001, k = 65) displayed no significant results. However, a more lenient threshold of p<0.05 showed large clusters with abnormally high FA values, and a striking spatial correspondence with the longitudinal FA increase along the SCR (Fig. 3). Specifically, ∼1,400 contiguous voxels were found to have abnormally high FA values in LATL patients and ∼1,100 voxels were found with abnormally high FA in RATL patients, always ipsilateral to the site of resection and along the SCR (Fig. 3). Another prediction of the Wallerian degeneration hypothesis is that larger resections would lead to larger FA increase as more fibers would be resected. To test this prediction, we correlated FA change in the SCR clusters with the respective resection ratios in LATL and RATL groups. A significant correlation was found in the left group (r = 0.61, p<0.05), but not in the right group (r = −0.45, p = 0.24). Thus, at least in the LATL group, the FA increase along the SCR was related to the resection size. Next, we checked whether larger resections impact more voxels or the magnitude of FA increase in the same voxels. Voxels with FA change beyond 1 STD were counted within the SCR cluster of each group. Correlation analyses showed a significant relationship between the number of voxels and the resection ratio in the LATL group (r = 0.63, p<0.05), but not in the RATL group (r = −0.43, p = 0.16).

Confirming the hypothesis of degeneration of crossing fibers, LATL moved toward more positive MO values in the left SCR cluster (ΔMO 95% CI = 0.02, 0.08) while controls did not change (ΔMO 95% CI = −0.04, 0.00). Similarly, RATL moved toward more positive values in the right SCR cluster (ΔMO 95% CI = 0.06, 0.15) while controls did not change (ΔMO 95% CI = −0.03, 0.01). The change in MO, in fact, yielded abnormally high MO values in both ATL groups post-surgery (LATL: T(22) = 3.38, p = 0.003; RATL: T(22) = 4.26, p<0.001) while, pre-surgery, the same patients were similar to controls (LTLE: T(22) = 0.62, p = 0.54; RTLE: T(22) = 0.34, p = 0.74).

### Evidence for plasticity in the contralateral language tracts (SLF and UNC) of LATL patients

The contralateral UNC and SLF increased FA only in LTLE patients. Whole-brain analysis did not show evidence of FA exceeding normal values in these clusters post-surgery. On the contrary, FA values were lower than in controls before surgery (for SLF, T(22) = −3.04, p = 0.006; for UNC, T(22) = −3.65, p = 0.001) and similar to controls after surgery (for SLF, T(22) = −0.98, p = 0.34; for UNC T(22) = −0.47, p = 0.65).

For both SLF and UNC, pre-to-post change in MO was similar between patients and controls (for SLF, T(22) = −0.47, p = 0.65; for UNC, T(22) = 0.21, p = 0.83); patients were similar to controls before surgery (for SLF, T(22) = −0.94, p = 0.36; for UNC, T(22) = −0.36, p = 0.73), and similar after surgery (for SLF, T(22) = −1.05, p = 0.31; for UNC, T(22) = −0.16, p = 0.87). In conclusion, FA normalization in the contralateral language tracts of LATL patients was driven by a decrease in λT, whereas λ∥ and MO were relatively unchanged.

### The role of interval to post-surgery scan

**Figure 4:**
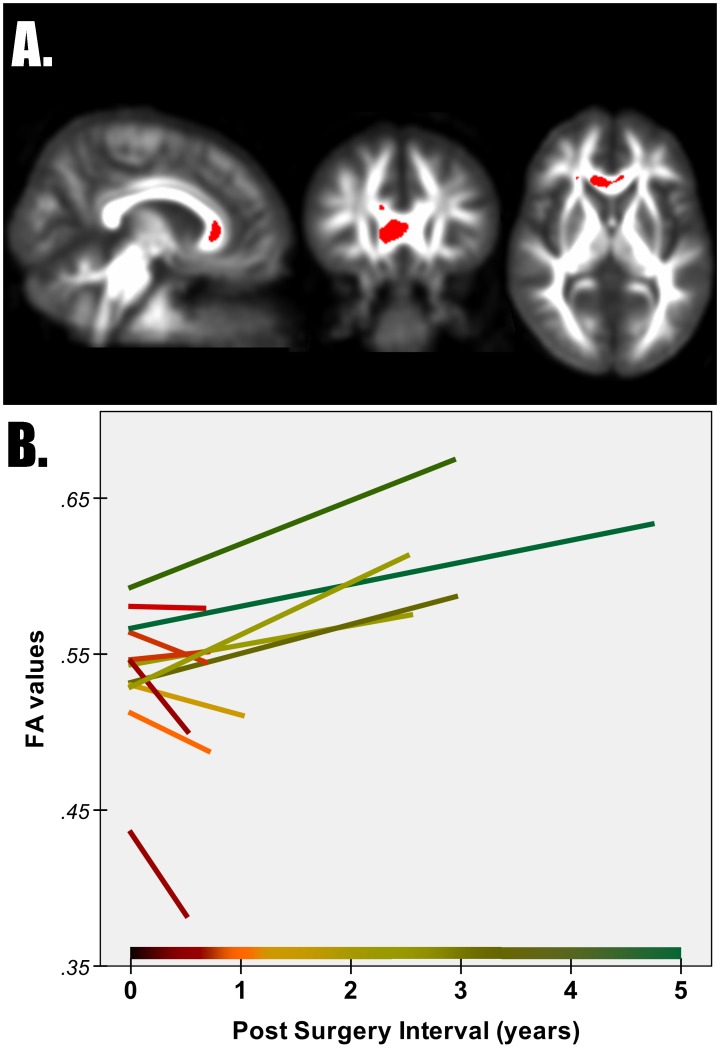
FA change with respect to post-surgery interval. Panel A shows FA change inversely related to post-surgery time interval in the genu of corpus callosum for LATL patients. Panel B shows raw FA values from the 12 LATL patients before (time 0) and after surgery in the same cluster shown in Panel A.

Since the two patient groups differed for the time interval between surgery and second scan, a separate whole-brain analysis was performed with this variable as covariate. The inclusion of the covariate did not change the reported pattern of post-surgery FA changes. The post-surgery interval, however, correlated with ΔFA in the genu of the corpus callosum of LATL patients. With increasing time intervals, FA changes in this cluster inverted from FA decreases to FA increases (Fig. 4).

### Neuropsychological correlates of FA increase

**Figure 5:**
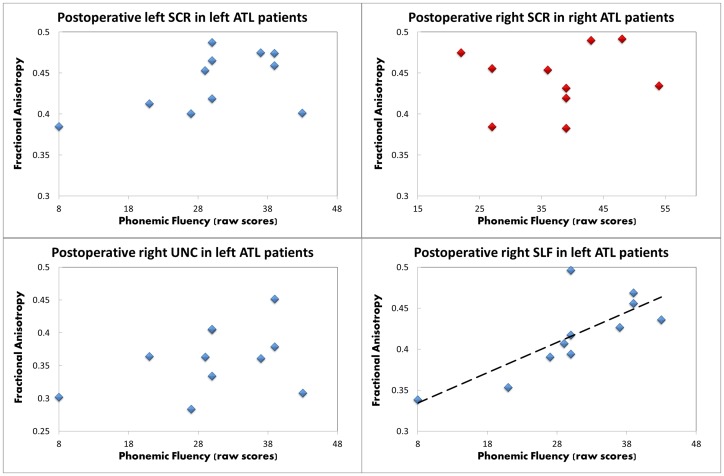
Postoperative relationship between phonemic fluency and FA values. The four clusters that increased FA in the two groups are shown in the four panels (blue = LATL group; red = RATL group). The regression line is placed for the significant correlation found in the right superior longitudinal fasciculus.

Correlations with verbal fluency scores were performed on 11 LATL and 10 RATL patients (excluding non-English speaking patients). In the LATL group, preoperative performance in phonemic fluency positively correlated with pre-surgical and post-surgical FA values in all three clusters showing an FA increase, namely the left SCR (pre-op: r = 0.65, p = 0.03; post-op: r = 0.83, p = 0.001), the right SLF (pre-op: r = 0.83, p = 0.001; post-op: r = 0.79, p = 0.002), and the right UNC (pre-op: r = 0.65, p = 0.02; post-op: r = 0.59, p = 0.03). Postoperative performance in phonemic fluency correlated only with FA of the right SLF, measured either before (r = 0.77, p = 0.003) or after surgery (r = 0.76, p = 0.003). Semantic fluency did not correlate with any cluster preoperatively (all p>0.1), but postoperatively correlated with FA of the right SLF, measured either before (r = 0.67, p = 0.02) or after surgery (r = 0.72, p = 0.009). In RATL patients, the right SCR cluster did not correlate with preoperative or postoperative fluency scores (all p>0.09). Fig. 5 displays correlations with postoperative phonemic fluency.

## Discussion

We investigated WM abnormalities in left and right TLE patients prior to surgery, then tracked WM changes after surgery, using repeated measures data on healthy-matched controls as a comparison group. This is one of the first attempts to investigate white matter changes in patients undergoing ATL, comparing the results with temporally matched changes in healthy controls.

Overall, both TLE groups showed diminished FA pre-surgery in comparison to the controls. However, this difference was more extensive in the left group (∼40,000 voxels in the LTLE group; ∼7,500 voxels in the RTLE group). Among the tracts affected exclusively in the LTLE group were the SLF bilaterally and the UNC contralaterally. In contrast, anterior parts of the internal/external capsules and corona radiata of the right hemisphere showed dramatic FA reduction in both TLE groups. These findings are in agreement with previous studies showing larger FA disruptions in left compared to right ATL patients [15], [48], and a particular sensibility of language tracts to left hemispheric pathology [4], [18].

Post-surgery, we found increases and decreases in FA in both patient groups. Regarding FA decreases, a symmetrical pattern was observed in LATL and RATL patients involving decreases in the fornix, SLF, UNC, callosum, inferior fronto-occipital and external capsule. Evidence of decreased FA post-surgery in ATL has been reported for all the above tracts in prior studies [25]–[30], [49]. In particular, all studies agree in finding FA disruptions in the fornix, which we consistently found to be the area of greatest diminishment for both ATL groups. In contrast, reports of FA increase have been limited, with only three studies reporting FA increase after surgery. Two of these studies have provided evidence for FA increase exclusively in LATL patients [25], [27], while more recently a third study found FA increases in both LATL and RATL patients, with increases in RATL developing later in time [30]. Given that FA increases could be a marker of neuroplastic processes in white matter, we focused our investigation on clusters that in showed FA increase, with the aim of distinguishing genuine neuroplasticity from changes that could be attributed to processes related to the degeneration of crossing fibers.

The most prominent FA increases post-surgery occurred in the superior corona radiata, both in left and right resected patients, ipsilateral to the resection. This finding is consistent with previous studies in epilepsy patients [25], [27], [30]. Furthermore, FA increase in this area was caused by a combined increase in parallel and decrease in perpendicular diffusivity, similar to the postoperative reports of Yogarajah et al. [27] and Winston et al. [30]. The commonality in the area of FA increase and the pattern of diffusion changes does suggest that the underlying mechanism of postoperative change is similar. While such increase was initially interpreted as a neuroplastic response related to reorganization of language networks [27], our results and the evidence recently published by Winston et al. [30] show that FA increases in the corona radiata occur both in left and right ATL group, a finding that suggests that these FA increases are not necessarily language related.

Rather than a genuine neuroplastic response, our findings suggest that FA increase along the SCR is caused by fiber degeneration in regions known to have crossing fibers [50], [51]. We found that FA values along the SCR, not only increase after surgery, but exceed the normative FA values obtained from control subjects. We also found that the mode of anisotropy, a measure previously used to investigate crossing fibers [38], [39], changed from normal before surgery to abnormally high after surgery, again suggesting the presence of fewer crossing fibers in the SCR region post-operatively. While these findings are suggestive of degeneration of crossing fibers, they do not fully exclude the possibility that a neuroplastic response may occur post-surgery. However, if a true neuroplastic response was the only reason behind the FA increase in the corona radiate, then quite substantial axonal changes must have occurred, with, for example, numerous new axons appearing in the area or existing axons displaying some kind of “hyper-myelination” that does not exist under normal conditions. This would need to have occurred in axons aligned in the same direction, ipsilateral to the resection, and in an area with crossing fibers. We view this possibility as highly unlikely and not supported by any evidence from lesion models in animals. A certain amount of use-dependent plasticity may, nonetheless, occur, in line with recent evidence on use-dependent WM reorganization (demonstrated in vitro [52] and in vivo in humans [53]–[59]). However, such plasticity is expected to be on the order of 1%–6% based on previous studies [54], [58], [59] while the typical increase observed in SCR after surgery is 8%–12% (see also, [27], [30]). Thus, our findings appear more in line with dramatic changes caused by degeneration of crossing fibers rather than a use-dependent hyper-myelination. Lastly, it is important to note that the corona radiata has been found to have abnormally high FA not only in postoperative epilepsy patients but also in patients with neurodegenerative diseases, such as Alzheimer and multiple sclerosis [38], [39], [60]. It is important to note that prior to entering the corona radiata, the same tracts have lower FA [61], consistent with the notion that fiber degeneration generates higher FA in the corona radiate, making FA an unreliable measure of neuroplasticity in this area. In summary, we considered both hypotheses regarding the FA increase in the SCR (i.e., genuine neuroplasticity versus degeneration of crossing fibers), and conclude that, overall, the degeneration hypothesis is more plausible.

Despite both LATL and RATL groups showing an FA increase in the ipsilateral corona radiata, one finding distinguishing these groups is the presence of a resection-size-dependent FA increase in LATL patients. It is tempting to argue that the relationship between resection size and FA increase in LATL patients might be related to reorganization of language networks occurring after dominant hemisphere resection. That is, larger resections may compel plasticity because of the adaptive value and the importance of maintaining language networks in the dominant hemisphere. On the other hand, larger resections may damage the left arcuate fasciculus and open the door to progressively larger loss of crossing fibers in the adjacent corona radiata. In this regard we note that the arcuate is more prominent in the left hemisphere [17], [62], [63], putting it more at risk with resective surgery. Recent evidence also suggests that the arcuate is longer than previously thought, with some fibers reaching not just the posterior but also the anterior temporal lobe [64]. A larger left arcuate that reaches anterior temporal areas increases the chance for resection-dependent Wallerian degeneration, eventually leading to a correlation between FA increase and resection size only in left ATL patients. It is also worth noting that FA increases appear to occur earlier in LATL than in RATL patients, as our findings and others have reported. For example, Yogarajah et al. [27] found limited increases in RATL patients ∼6 months post-surgery, while we found prominent and comparable increases in LATL and RATL patients over one year post-surgery. Winston et al. [30] further investigated the role of post-surgery interval by obtaining multiple time points after resection, and found that WM changes in LATL patients occur predominantly in the first months post-surgery, while changes in RATL patients continue throughout the entire first year post-op. The reason for an ipsilateral resection dependent increase in left but not right ATL, and why this might be present earlier in LATL patients, is not clear. Ultimately, we consider the correlation of FA increase with resection size to be equivocal with regards to the competing hypotheses of plasticity versus degeneration of crossing fibers. As a result of this, we do not interpret the correlation to reflect genuine neuroplasticity in the corona radiata.

Regarding our findings for the SCR, another interpretation could be that the resected hemisphere physically shifts in the resected cavity (*ex-vacuo* movement) allowing more parallel fibers to be measured on each voxel. However, the finding that only left ATL patients showed a resection-dependent FA increase, despite having similar resection size with right ATL patients (or, even more, a trend of larger resections in right ATL), suggests that a hemispheric shift is not the main reason behind these findings.

Beside the FA increase in ipsilateral SCR clusters, which might be affected by changes in the amount of crossing fibers, FA increase was observed in two additional clusters in contralateral language tracts of LATL patients: superior longitudinal and uncinate fasciculi. In these clusters, FA normalized after surgery and no evidence was found to indicate a substantive effect of crossing fibers. Different from the SCR clusters, FA increase in these clusters was driven, not by combined changes in parallel and perpendicular diffusivities, but mostly by a decrease in perpendicular diffusivity, a finding that suggests a different type of white matter reorganization has occurred in the contralateral language tracts compared to the ipsilateral SCR clusters. Decreased perpendicular diffusivity may indicate increased myelination [36] but is not specific only to this process [34], [35]. It is this difference in the pattern of change in parallel and perpendicular diffusivities that suggests different underlying processes are at work in the ipsilateral SCR compared to the contralateral SLF and UNC.

The relationship of FA with behavioral performance may provide an important indicator of the functional adaptation occurring pre- to post-surgery. Only the study of Yogarajah et al. [27] investigated the relationship of FA increase to behavioral performance. They found that FA increases in the ipsilateral hemisphere (including the SCR) correlated with fluency scores, and considered this as suggestive of intra-hemispheric language reorganization. Our data suggest a different pattern of potential reorganization. Preoperative phonemic fluency in our LATL patients significantly correlated with FA values of all three clusters that increased FA (left SCR, right UNC, right SLF). However, postoperative phonemic fluency correlated only with FA of the right SLF. We also observed the emergence of a correlation of semantic fluency with right SLF. Given the relationship of all three clusters with language performance before surgery but only the right SLF after surgery, our data is consistent with the possibility that an adaptive inter-hemispheric shift took place with larger involvement of the contralateral non-dominant hemisphere. Supporting this interpretation, several fMRI studies have reported a functional shift of language processes in the non-dominant hemisphere post-ATL [65]–[68]. A recent study, in particular, investigated epilepsy patients before and after ATL surgery, showing a shift of fMRI activations post-surgery into a more bilateral pattern [65]. Importantly, the same patients showed increased functional connectivity between left and right frontal lobes, further contributing to the notion of inter-hemispheric shift of language processes [65]. As seizure control may be an important factor increasing the likelihood of adaptive reorganization, we should note that a high proportion of LATL patients in our study experienced a good seizure outcome (e.g., 83% with Engel class I). Our results provide the first evidence for structural reorganization of language tracts in the non-dominant hemisphere, a process which may implement the compensatory shifts in task-related or resting-state activation that has been reported by our lab as well as others [65], [69], [70].

The results presented here, though providing new evidence, are limited by several factors of note. First, the sample size in our study is not optimal to apply robust statistical analyses (i.e. FWE corrected), increasing the possibility of false positives and decreasing the ability to discriminate more subtle FA changes. However, we did perform analyses to detect outliers and never were the results driven by single isolated subjects. Moreover, our findings were often consistent with previous literature. Second, while the changes observed in “mode of anisotropy” were both significant and important, there remains much that is unknown about this measure. For example, it is not clear whether the limitations affecting parallel and perpendicular diffusivities in areas with crossing fibers [71], affect MO as well, a limitation that would alter its biological significance as a measure of crossing fibers [38], [39]. In general, “mode” has not been studied thoroughly and has not been frequently used in the literature, and our interpretation does not solely rely on it. A third limitation may arise from a slightly different proportion of patients with MTS in our two TLE groups, though statistically the groups were equivalent (six MTS in LTLE, three MTS in RTLE). Several studies have shown that the presence or absence of MTS produces distinct patterns of white matter abnormality [2], [7], [8], [72], [73]. In this regard, an individual inspection of the FA results in the LATL group did not show a difference based on MTS. Fourth, gender distribution was different between our groups, raising a concern about gender related white matter changes [74]. However, the sample size available was too small to allow for a proper control of this variable. Lastly, the medication pattern in our sample varied too much to allow for any medication subgroup comparisons. The majority of our patients, however, did not change medication regimens pre-to-post surgery, suggesting this factor did not affect the pre-to-post FA changes we report.

## Conclusions

The biological and functional significance of FA increases is of great importance for understanding the potential for white matter reorganization following surgery. By including matched controls in a longitudinal clinical study we were able to distinguish changes in water diffusion properties potentially reflective of adaptive neuroplastic responses from changes that more likely reflect degeneration in specific regions that have crossing fibers. Our data confirmed recent evidence by Winston et al. [30] who showed that FA increases do occur following both left and right ATL resection. We extend these findings by clarifying the nature of the various change processes that can occur after ATL, providing evidence that Wallerian degeneration likely explains the FA increase observed in some areas (e.g., SCR ipsilateral to the resection). Importantly, our results stand in contrast to prior work [27] that showed adaptive neuroplasticity, as measured by FA increase, only in the dominant hemisphere. Our evidence suggests that structural white matter reorganization of language tracts may occur in the contralateral, non-dominant hemisphere, following resection of the dominant anterior temporal lobe. Lastly, our data also provide some suggestion that the changes observed post-surgery may be a function of the time interval post-resection when the FA measures are collected, a finding consistent with previous literature [25], [30]. This raises a quite speculative possibility that different WM regions reorganize and generate a neuroplastic response at different rates and times post-surgery, perhaps related to the functional day-to-day importance of the gray matter regions to which they connect (e.g., expressive language). This study has implications for determining functional risk in candidates for temporal lobe surgery, as our data suggest that left TLE patients, despite showing initially more extensive structural orientation damage, have the potential for contralateral WM structural reorganization in language tracts.

## Supporting Information

Table S1 **Demographic details of all subjects.**
(DOC)

Table S2 **Tracts with lower FA in patients compared to controls, before surgery.**
(DOC)

Table S3 **Tracts with FA decrease in patients compared to controls, after surgery.**
(DOC)

Table S4 **Tracts with FA increase in patients compared to controls, after surgery.**
(DOC)

File S1 **Joint histograms of the non-linear longitudinal registration of FA maps.**
(DOC)
