# Supplementary material for: Distinct Types of White Matter Changes Are Observed after Anterior Temporal Lobectomy in Epilepsy
Source: PLoS One. 2014 Aug 4;9(8):e104211. doi: 10.1371/journal.pone.0104211 (PMC4121328; doi:10.1371/journal.pone.0104211)
Supplement: Table S1 — Demographic details of all subjects. (DOC) [file pone.0104211.s001.doc]

**Table S1**: Demographic details of all subjects in the study. Outcome class is based on Engel classification. Abbreviations: SPS=simple partial seizures; CPS=complex partial seziures; GTCS=generalized tonic clonic seizures; secGTCS=CPS with secondary generalization; MTS=mesial temporal sclerosis. MEDICATION: Kep=keppra; Trilep=trileptal; Lamic=lamictal; Psych=psychotropic; Depa=Depakote; Phenob=phenobarbital; Teg=tegretol; Zonis=zonisamide; Vimp=vimpat; Topo=topomax; Gabit=gabitril.

| **N** | **Group** | **Age** | **Gender** | **Age Onset** | **Epil. Duration (yrs)** | **Handedness** | **fMRI laterality** | **Post-surg. Interval (yrs)** | **Resect. Vol. (mm3)** | **Cereb. Vol. (L)** | **Resection ratio (%)** | **Seizure type** | **MRI** | **PET** | **Outcome Class** | **Pre-surg. medication** | **Post-surg. medication** |
| --- | --- | --- | --- | --- | --- | --- | --- | --- | --- | --- | --- | --- | --- | --- | --- | --- | --- |
| 1 | LTLE | 56 | M | 16 | 40 | RH |  | 2.54 | 7053 | 1.27 | 0.55 | SPS | L. Temp. encefalomalacia. | L. temp. hypo. | 1 | Kep., Trilept. | Trilep. |
| 2 | LTLE | 44 | F | 3 | 41 | RH | -0.86 | 0.70 | 11980 | 1.26 | 0.95 | CPS; rare GTCS | L. MTS | R. MTL hypo. | 1 | Kep., Psych. | Lamic., Psych. |
| 3 | LTLE | 60 | F | 53 | 7 | RH |  | 2.95 | 33181 | 1.29 | 2.57 | SPS/CPS | normal | L temp. hypo. | 1 | Kep. | Kep. |
| 4 | LTLE | 36 | M | 20 | 16 | RH |  | 2.93 | 27814 | 1.37 | 2.03 | CPS | normal | L temp. hypo. | 1 | Lamic., Psych. | Lamic., Kep., Psych. |
| 5 | LTLE | 42 | F | 5 | 37 | RH | -0.94 | 0.50 | 8942 | 1.20 | 0.75 | SPS/CPS | L. MTS + L. Temp. atrophy | L. MTL hypo. | 3 | Kep., Depa., Phenob. | Kep., Psych. |
| 6 | LTLE | 42 | F | 38 | 4 | RH |  | 0.68 | 21816 | 1.24 | 1.76 | CPS | L. MTS | L. MTL hypo. | 1 | Lamic. | Lamic. |
| 7 | LTLE | 60 | F | 13 | 47 | RH | -0.65 | 1.01 | 13315 | 1.22 | 1.09 | CPS | L. MTS | B/L temp. hypo. | 1 | Phenob., Teg. | Phenob., Teg. |
| 8 | LTLE | 36 | F | 2 | 34 | LH | -0.82 | 0.51 | 17791 | 1.16 | 1.53 | CPS | normal | L. MTL hypo. | 4 | Lamic., Psych. | Teg., Vimp., Zonis., Psych. |
| 9 | LTLE | 52 | F | 13 | 39 | RH | -0.42 | 0.70 | 16605 | 1.15 | 1.45 | CPS | L. MTS |  | 1 | Kep., Trilept. | Teg. |
| 10 | LTLE | 25 | F | 17 | 7 | RH | -0.72 | 0.66 | 24518 | 1.28 | 1.91 | CPS with secGTCS | L Ant. Temp. dysplasia | B/L temp. hypo. | 1 | Vimp., Psych. | Vimp., Psych. |
| 11 | LTLE | 35 | F | 14 | 21 | RH | -0.86 | 4.74 | 22421 | 1.39 | 1.61 | CPS | L. MTS |  | 1 | Lamic., Zonis. | Lamic. |
| 12 | LTLE | 34 | F | 19 | 15 | RH | -0.92 | 2.51 | 28113 | 1.15 | 2.44 | SPS/CPS | normal | R. MTL hypo. | 1 | Trilep., Psych. | Trilep., Psych. |
| 13 | RTLE | 33 | F | 2 | 31 | RH |  | 1.78 | 34394 | 1.17 | 2.95 | CPS/rare GTCS | R. MTS | normal | 1 | Trilep., Psych | Zonis., Psych. |
| 14 | RTLE | 30 | F | 10 | 20 | RH |  | 1.47 | 16356 | 0.95 | 1.73 | CPS | normal | R. temp. hypo. | 5 | Kep., Psych. | Trilep., Psych. |
| 15 | RTLE | 55 | M | 5 | 50 | RH | -0.83 | 0.44 | 24624 | 1.36 | 1.81 | CPS; rare secGTCS | R. MTS | R. temp. hypo. | 1 | Kep. | Kep., Teg. |
| 16 | RTLE | 59 | M | 27 | 32 | RH |  | 0.78 | 17198 | 1.19 | 1.44 | CPS | normal | R. temp. hypo. | 1 | Lamic. | Lamic., Psych. |
| 17 | RTLE | 28 | M | 16 | 12 | RH | -0.73 | 0.71 | 28079 | 1.59 | 1.77 | CPS | normal | normal | 1 | Depak. | Kep., Trilep. |
| 18 | RTLE | 25 | M | 19 | 6 | RH | -0.93 | 0.68 | 25327 | 1.43 | 1.77 | CPS/SPS | normal | R. temp. hypo. | 1 | Kep., Depak. | Kep. |
| 19 | RTLE | 65 | M | 20 | 45 | AMB | 0.59 | 0.68 | 26947 | 1.69 | 1.59 | CPS | R. MTS | R. temp. hypo. | 1 | Topo., Psych. | Topo., Psych |
| 20 | RTLE | 31 | F | 25 | 5 | LH | -0.83 | 0.41 | 17138 | 1.19 | 1.44 | CPS/SPS | normal | R. temp. hypo. | 4 | Vimp., Zonis. | Gabit., Psych., |
| 21 | RTLE | 39 | M | 35 | 4 | RH | -0.91 | 0.47 | 17594 | 1.50 | 1.18 | CPS | normal | R. temp. hypo. | 1 | Vimp., Lamic. | Vimp., Lamic., Psych. |
| 22 | RTLE | 30 | M | 16 | 14 | RH | -0.88 | 0.55 | 40933 | 1.43 | 2.87 | CPS/GTCS | R. Temp. encephalomalacia |  | 1 | Phenov. | Phenob., Psych. |
| 23 | RTLE | 47 | F | 34 | 13 | LH | -0.56 | 0.91 | 56612 | 1.24 | 4.58 | CPS | normal | R. temp. hypo. | 1 | Vimp. | Vimp. |
| 24 | RTLE | 52 | F | 11 | 41 | RH | -0.84 | 0.63 | 19344 | 1.23 | 1.57 | CPS/secGTCS | normal | R. temp. hypo. | 1 | Lamic., Teg. | Lamic., Teg |
| 25 | control | 42 | M |  |  | RH | -0.85 |  |  |  |  |  |  |  |  |  |  |
| 26 | control | 40 | M |  |  | RH | -0.81 |  |  |  |  |  |  |  |  |  |  |
| 27 | control | 50 | M |  |  | RH | -0.87 |  |  |  |  |  |  |  |  |  |  |
| 28 | control | 40 | M |  |  | RH | -0.76 |  |  |  |  |  |  |  |  |  |  |
| 29 | control | 44 | M |  |  | RH | -0.9 |  |  |  |  |  |  |  |  |  |  |
| 30 | control | 43 | F |  |  | RH | -0.77 |  |  |  |  |  |  |  |  |  |  |
| 31 | control | 61 | F |  |  | LH | -0.53 |  |  |  |  |  |  |  |  |  |  |
| 32 | control | 59 | F |  |  | RH | -0.96 |  |  |  |  |  |  |  |  |  |  |
| 33 | control | 57 | M |  |  | RH | -0.82 |  |  |  |  |  |  |  |  |  |  |
| 34 | control | 48 | M |  |  | RH | -0.84 |  |  |  |  |  |  |  |  |  |  |
| 35 | control | 60 | M |  |  | RH | -0.86 |  |  |  |  |  |  |  |  |  |  |
| 36 | control | 43 | M |  |  | RH | -0.67 |  |  |  |  |  |  |  |  |  |  |
