# Supplementary material for: Distinct Types of White Matter Changes Are Observed after Anterior Temporal Lobectomy in Epilepsy
Source: PLoS One. 2014 Aug 4;9(8):e104211. doi: 10.1371/journal.pone.0104211 (PMC4121328; doi:10.1371/journal.pone.0104211)
Supplement: Table S2 — Tracts with lower FA in patients compared to controls, before surgery. (DOC) [file pone.0104211.s002.doc]

Table S2: Tracts with lower FA in patients compared to controls, before surgery.

Values indicate voxel count and percentage of the tract involved. Tstat = average t-value; Pval = average p-value in logarithmic scale, eg. 4 = 0.0001; Dcoh = average effect size, Cohen’s D. Conjunction analysis shows areas that are affected both in left and right TLE patients. Abreviations: Fx/ST=fornix/stria terminalis; Hippo=hippocampus; CGH=cingulum of hippocampus / parahippocampal; CGC=cingulate of cingulate gyrus; GCC=genu of callosum; BCC=body of callosum; SCC=splenium of callosum; SFO=superior fronto-occipital fasciculus; SLF=superior longitudinal fasciculus; UNC=uncinate fasciculus; IFO=inferior fronto-occipital fasciculus; SS=sagittal stratum, which includes inferior longitidinal fasciculus and inferior fronto-occipital fasciculus; CST=corticospinal tract; EC=external capsule; ALIC=anterior limb of internal capsule; PLIC=posterior limb of internal capsule; RLIC=retrolenticular part of internal capsule; ACR=anterior corona radiata; SCRsuperior corona radiata; PCR=posterior corona radiata; PTR=posterior thalamic radiation; Temporal WM=inferior/superior/middle temporal white matter; Parietal WM=superior/postcentral/angular/supramarginal parietal white matter; Frontal WM=superior/middle/inferior/precentral frontal white matter.

| **PRE-Surgery**  **reduced FA** | **Left TLE (n=12)** | | **Right TLE (n=12)** | | **Conj. LTLE and RTLE** | |
| --- | --- | --- | --- | --- | --- | --- |
|  | Ipsilat. | Contralat. | Ipsilat. | Contralat. | Left | Right |
| limbic |  |  |  |  |  |  |
| Fx/ST  Tstat / Pval / Dcoh | 130 (11%)  3.62 / 3.31 / 1.26 | 450 (39%)  3.94 / 3.70 / 1.37 |  |  |  |  |
| Hippo  Tstat / Pval / Dcoh | 353 (7%)  4.04 / 3.83 / 1.41 | 406 (9%)  3.82 / 3.56 / 1.33 |  | 44 (1%)  3.68 / 3.38 / 1.28 | 31 (1%)  3.75 / 3.47 / 1.30 |  |
| CGH  Tstat / Pval / Dcoh | 336 (37%)  4.54 / 4.46 / 1.58 | 329 (28%)  4.07 / 3.87 / 1.42 |  | 119 (13%)  4.14 / 3.95 / 1.44 | 118 (13%)  4.14 / 3.96 / 1.44 |  |
| CGC  Tstat / Pval / Dcoh | 1916 (50%)  4.00 / 3.78 / 1.39 | 1924 (63%)  4.06 / 3.86 / 1.41 | 884 (29%)  3.83 / 3.57 / 1.33 | 426 (11%)  3.79 / 3.52 / 1.32 | 342 (9%)  3.66 / 3.36 / 1.27 | 726 (24%)  3.74 / 3.46 / 1.30 |
| Corpus callosum |  |  |  |  |  |  |
| GCC  Tstat / Pval / Dcoh | 522 (14%)  3.77 / 3.50 / 1.31 | 824 (18%)  3.71 / 3.43 / 1.29 | 437 (10%)  3.67 / 3.37 / 1.28 | 18 (0%)  3.39 / 3.04 / 1.18 | 10 (0%)  3.39 / 3.03 / 1.18 | 295 (6%)  3.62 / 3.32 / 1.26 |
| BCC  Tstat / Pval / Dcoh | 2212 (43%)  3.95 / 3.72 / 1.37 | 2321 (39%)  3.98 / 3.75 / 1.38 | 148 (2%)  3.72 / 3.43 / 1.29 | 35 (1%)  3.68 / 3.39 / 1.28 | 35 (1%)  3.68 / 3.39 / 1.28 | 147 (2%)  3.72 / 3.43 / 1.29 |
| SCC  Tstat / Pval / Dcoh | 1541 (25%)  4.00 / 3.78 / 1.39 | 410 (6%)  3.72 / 3.44 / 1.30 |  | 388 (6%)  3.76 / 3.48 / 1.31 | 370 (6%)  3.77 / 3.50 / 1.31 |  |
| major tracts |  |  |  |  |  |  |
| SFO  Tstat / Pval / Dcoh | 72 (22%)  3.67 / 3.37 / 1.28 | 176 (79%)  4.08 / 3.88 / 1.42 | 38 (17%)  3.56 / 3.24 / 1.24 |  |  | 38 (17%)  3.56 / 3.24 / 1.24 |
| SLF  Tstat / Pval / Dcoh | 474 (10%)  3.78 / 3.51 / 1.32 | 589 (11%)  3.79 / 3.52 / 1.32 |  |  |  |  |
| UNC  Tstat / Pval / Dcoh |  | 60 (32%)  3.97 / 3.74 / 1.38 |  |  |  |  |
| IFO  Tstat / Pval / Dcoh | 109 (7%)  3.91 / 3.67 / 1.36 | 1197 (69%)  4.51 / 4.42 / 1.57 | 725 (42%)  3.88 / 3.64 / 1.35 |  |  | 558 (32%)  3.89 / 3.65 / 1.36 |
| SS  Tstat / Pval / Dcoh | 1065 (43%)  4.14 / 3.96 / 1.44 | 673 (27%)  3.77 / 3.50 / 1.31 | 174 (7%)  3.91 / 3.67 / 1.36 | 330 (13%)  4.29 / 4.14 / 1.49 | 259 (10%)  4.09 / 3.89 / 1.42 | 44 (2%)  3.46 / 3.12 / 1.20 |
| CST  Tstat / Pval / Dcoh | 18 (1%)  3.59 / 3.28 / 1.25 | 141 (10%)  3.92 / 3.68 / 1.36 |  |  |  |  |
| EC  Tstat / Pval / Dcoh | 595 (25%)  4.02 / 3.80 / 1.40 | 1947 (62%)  4.63 / 4.57 / 1.61 | 500 (16%)  4.06 / 3.86 / 1.41 |  |  | 494 (16%)  4.06 / 3.86 / 1.41 |
| internal capsule |  |  |  |  |  |  |
| ALIC  Tstat / Pval / Dcoh | 1745 (64%)  4.03 / 3.82 / 1.40 | 2189 (90%)  4.75 / 4.73 / 1.65 | 594 (24%)  4.16 / 3.99 / 1.45 | 185 (7%)  3.65 / 3.36 / 1.27 | 129 (5%)  3.69 / 3.40 / 1.28 | 576 (24%)  4.18 / 4.00 / 1.45 |
| PLIC  Tstat / Pval / Dcoh | 2266 (63%)  4.21 / 4.05 / 1.47 | 1348 (39%)  4.22 / 4.05 / 1.47 | 41 (1%)  3.55 / 3.23 / 1.24 | 35 (1%)  3.48 / 3.14 / 1.21 | 35 (1%)  3.48 / 3.14 / 1.21 | 41 (1%)  3.55 / 3.23 / 1.24 |
| RLIC  Tstat / Pval / Dcoh | 817 (41%)  3.95 / 3.71 / 1.37 | 609 (26%)  3.77 / 3.50 / 1.31 |  |  |  |  |
| corona radiata |  |  |  |  |  |  |
| ACR  Tstat / Pval / Dcoh | 538 (8%)  4.01 / 3.79 / 1.39 | 1130 (15%)  4.37 / 4.25 / 1.52 | 603 (8%)  3.97 / 3.75 / 1.38 | 99 (1%)  3.71 / 3.42 / 1.29 |  | 519 (7%)  3.99 / 3.77 / 1.39 |
| SCR  Tstat / Pval / Dcoh | 523 (5%)  3.75 / 3.47 / 1.30 | 1407 (14%)  4.09 / 3.89 / 1.42 | 377 (4%)  3.67 / 3.38 / 1.28 |  |  | 290 (3%)  3.69 / 3.40 / 1.29 |
| PCR  Tstat / Pval / Dcoh | 43 (3%)  3.80 / 3.53 / 1.32 | 183 (10%)  4.22 / 4.05 / 1.47 |  |  |  |  |
| PTR  Tstat / Pval / Dcoh | 135 (2%)  3.55 / 3.23 / 1.24 | 769 (14%)  3.79 / 3.52 / 1.32 | 35 (1%)  3.71 / 3.43 / 1.29 |  |  | 28 (1%)  3.75 / 3.48 / 1.31 |
| general WM |  |  |  |  |  |  |
| Temporal WM  Tstat / Pval / Dcoh | 1250 (14%)  4.11 / 3.92 / 1.43 | 1303 (12%)  3.95 / 3.71 / 1.37 | 621 (6%)  3.67 / 3.38 / 1.28 | 246 (3%)  3.70 / 3.41 / 1.29 | 225 (3%)  3.71 / 3.42 / 1.29 | 378 (3%)  3.67 / 3.38 / 1.28 |
| Parietal WM  Tstat / Pval / Dcoh | 976 (9%)  3.83 / 3.57 / 1.33 | 739 (8%)  3.96 / 3.73 / 1.38 | 191 (2%)  3.90 / 3.66 / 1.36 |  |  | 171 (2%)  3.80 / 3.54 / 1.32 |
| Frontal WM  Tstat / Pval / Dcoh | 488 (2%)  3.83 / 3.57 / 1.33 | 1496 (6%)  3.83 / 3.57 / 1.33 | 250 (1%)  3.88 / 3.64 / 1.35 | 76 (0%)  3.74 / 3.46 / 1.30 | 72 (0%)  3.73 / 3.45 / 1.30 | 146 (1%)  3.77 / 3.50 / 1.31 |
