# Supplementary material for: Distinct Types of White Matter Changes Are Observed after Anterior Temporal Lobectomy in Epilepsy
Source: PLoS One. 2014 Aug 4;9(8):e104211. doi: 10.1371/journal.pone.0104211 (PMC4121328; doi:10.1371/journal.pone.0104211)
Supplement: Table S3 — Tracts with FA decrease in patients compared to controls, after surgery. (DOC) [file pone.0104211.s003.doc]

Table S3: Tracts with FA decrease in patients compared to controls, after surgery.

Values indicate voxel count and percentage of the tract involved. Tstat = average t-value; Pval = average p-value in logarithmic scale, eg. 4 = 0.0001; Dcoh = average effect size, Cohen’s D. Conjunction analysis shows areas that are affected both in left and right ATL patients. Abbreviations: FX=body of fornix; Fx/ST=fornix/stria terminalis; Hippo=hippocampus; CGH=cingulum of hippocampus / parahippocampal; CGC=cingulate of cingulate gyrus; GCC=genu of callosum; BCC=body of callosum; SCC=splenium of callosum; SFO=superior fronto-occipital fasciculus; SLF=superior longitudinal fasciculus; UNC=uncinate fasciculus; IFO=inferior fronto-occipital fasciculus; SS=sagittal stratum, which includes inferior longitidinal fasciculus and inferior fronto-occipital fasciculus; CST=corticospinal tract; EC=external capsule; ALIC=anterior limb of internal capsule; PLIC=posterior limb of internal capsule; RLIC=retrolenticular part of internal capsule; ACR=anterior corona radiata; SCRsuperior corona radiata; PCR=posterior corona radiata; PTR=posterior thalamic radiation; Temporal WM=inferior/superior/middle temporal white matter; Parietal WM=superior/postcentral/angular/supramarginal parietal white matter; Frontal WM=superior/middle/inferior/precentral frontal white matter.

| **POST-Surgery**  **FA decrease** | **Left ATL (n=12)** | | **Right ATL (n=12)** | | **Conj. LATL and RATL** | |
| --- | --- | --- | --- | --- | --- | --- |
|  | Ipsilat. | Contralat. | Ipsilat. | Contralat. | Left | Right |
| limbic |  |  |  |  |  |  |
| FX  Tstat / Pval / Dcoh | 87 (100%)  5.85 /6.11 / 2.04 | 205 (71%)  4.52 / 4.44 / 1.57 | 279 (97%)  5.56 / 5.75 / 1.94 | 75 (86%)  4.77 / 4.75 / 1.66 | 75 (86%)  4.77 / 4.75 / 1.66 | 205 (71%)  4.49 / 4.40 / 1.56 |
| Fx/ST  Tstat / Pval / Dcoh | 277 (23%)  4.03 / 3.82 / 1.40 |  | 497 (43%)  4.75 / 4.73 / 1.65 |  |  |  |
| CGH  Tstat / Pval / Dcoh | 66 (7%)  3.82 / 3.55 / 1.33 |  | 152 (13%)  4.24 / 4.09 / 1.48 |  |  |  |
| CGC  Tstat / Pval / Dcoh | 83 (2%)  3.88 / 3.63 / 1.35 |  | 88 (3%)  4.62 / 4.56 / 1.61 |  |  |  |
| Corpus callosum |  |  |  |  |  |  |
| GCC  Tstat / Pval / Dcoh | 485 (13%)  4.28 / 4.13 / 1.49 |  | 388 (8%)  4.08 / 3.88 / 1.42 |  |  |  |
| BCC  Tstat / Pval / Dcoh | 881 (17%)  4.04 / 3.83 / 1.41 |  | 1926 (32%)  4.12 / 3.93 / 1.43 |  |  |  |
| SCC  Tstat / Pval / Dcoh | 229 (4%)  3.88 / 3.64 / 1.35 |  | 1405 (21%)  4.43 / 4.32 / 1.54 |  |  |  |
| major tracts |  |  |  |  |  |  |
| SLF  Tstat / Pval / Dcoh | 539 (12%)  4.26 / 4.12 / 1.48 |  | 131 (2%)  3.99 / 3.77 / 1.39 |  |  |  |
| UNC  Tstat / Pval / Dcoh | 235 (97%)  7.40 / 7.99 / 2.58 |  | 183 (99%)  8.74 / 9.58 / 3.04 |  |  |  |
| IFO  Tstat / Pval / Dcoh | 631 (43%)  6.95 / 7.46 / 2.42 |  | 705 (41%)  6.19 / 6.51 / 2.16 |  |  |  |
| SS  Tstat / Pval / Dcoh | 326 (13%)  4.97 / 5.00 / 1.73 |  | 304 (12%)  6.07 / 6.36 / 2.11 |  |  |  |
| EC  Tstat / Pval / Dcoh | 88 (4%)  4.70 / 4.66 / 1.64 |  | 19 (1%)  3.88 / 3.63 / 1.35 |  |  |  |
| internal capsule |  |  |  |  |  |  |
| PLIC  Tstat / Pval / Dcoh |  |  | 58 (2%)  4.24 / 4.09 / 1.48 |  |  |  |
| RLIC  Tstat / Pval / Dcoh |  |  | 20 (1%)  3.97 / 3.74 / 1.38 |  |  |  |
| corona radiata |  |  |  |  |  |  |
| ACR Tstat / Pval / Dcoh | 28 (0%)  3.70 / 3.41 / 1.29 |  |  |  |  |  |
| SCR  Tstat / Pval / Dcoh |  |  | 64 (1%)  3.78 / 3.51 / 1.32 |  |  |  |
| PTR  Tstat / Pval / Dcoh | 67 (1%)  3.92 / 3.68/ 1.36 |  | 401 (7%)  3.84 / 3.59 / 1.34 |  |  |  |
| general WM |  |  |  |  |  |  |
| Parietal WM  Tstat / Pval / Dcoh | 900 (9%)  4.37 / 4.24 / 1.52 |  | 164 (2%)  4.07 / 3.87 / 1.42 |  |  |  |
| Frontal WM  Tstat / Pval / Dcoh | 1489 (7%)  4.32 / 4.18 / 1.50 |  | 651 (3%)  4.21 / 4.04 / 1.46 |  |  |  |
