# Supplementary material for: Distinct Types of White Matter Changes Are Observed after Anterior Temporal Lobectomy in Epilepsy
Source: PLoS One. 2014 Aug 4;9(8):e104211. doi: 10.1371/journal.pone.0104211 (PMC4121328; doi:10.1371/journal.pone.0104211)
Supplement: Table S4 — Tracts with FA increase in patients compared to controls, after surgery. (DOC) [file pone.0104211.s004.doc]

Table S4: Tracts with FA increase in patients compared to controls, after surgery.

Values indicate voxel count and percentage of the tract involved. Tstat = average t-value; Pval = average p-value in logarithmic scale, eg. 4 = 0.0001; Dcoh = average effect size, Cohen’s D. Conjunction analysis shows areas that are affected both in left and right ATL patients. Abbreviations: FX=body of fornix; Fx/ST=fornix/stria terminalis; Hippo=hippocampus; CGH=cingulum of hippocampus / parahippocampal; CGC=cingulate of cingulate gyrus; GCC=genu of callosum; BCC=body of callosum; SCC=splenium of callosum; SFO=superior fronto-occipital fasciculus; SLF=superior longitudinal fasciculus; UNC=uncinate fasciculus; IFO=inferior fronto-occipital fasciculus; SS=sagittal stratum, which includes inferior longitidinal fasciculus and inferior fronto-occipital fasciculus; CST=corticospinal tract; EC=external capsule; ALIC=anterior limb of internal capsule; PLIC=posterior limb of internal capsule; RLIC=retrolenticular part of internal capsule; ACR=anterior corona radiata; SCRsuperior corona radiata; PCR=posterior corona radiata; PTR=posterior thalamic radiation; Temporal WM=inferior/superior/middle temporal white matter; Parietal WM=superior/postcentral/angular/supramarginal parietal white matter; Frontal WM=superior/middle/inferior/precentral frontal white matter.

| **POST-Surgery**  **FA increase** | **Left ATL (n=12)** | | **Right ATL (n=12)** | | **Conj. LATL and RATL** | |
| --- | --- | --- | --- | --- | --- | --- |
|  | Ipsilat. | Contralat. | Ipsilat. | Contralat. | Left | Right |
| Limbic |  |  |  |  |  |  |
| CGC  Tstat / Pval / Dcoh |  | 30 (1%)  3.74 / 3.46 / 1.30 |  |  |  |  |
| Corpus callosum |  |  |  |  |  |  |
| GCC  Tstat / Pval / Dcoh |  | 263 (6%)  3.88 / 3.64 / 1.35 |  |  |  |  |
| major tracts |  |  |  |  |  |  |
| SFO  Tstat / Pval / Dcoh | 25 (8%)  4.03 / 3.82 / 1.40 | 14 (6%)  3.56 / 3.25 / 1.24 | 38 (17%)  3.92 / 3.69 / 1.37 |  |  |  |
| SLF  Tstat / Pval / Dcoh | 179 (4%)  3.73 / 3.45 / 1.30 | 306 (6%)  3.95 / 3.72 / 1.37 | 187 (4%)  3.94 / 3.71 / 1.37 |  |  |  |
| UNC  Tstat / Pval / Dcoh |  | 16 (9%)  3.59 / 3.28 / 1.25 |  |  |  |  |
| EC  Tstat / Pval / Dcoh | 83 (3%)  4.24 / 4.08 / 1.48 | 68 (2%)  3.62 / 3.32 / 1.26 | 111 (4%)  3.97 / 3.74 / 1.38 |  |  |  |
| internal capsule |  |  |  |  |  |  |
| ALIC  Tstat / Pval / Dcoh | 104 (4%)  4.09 / 3.90 / 1.42 | 42 (2%)  3.65 / 3.36 / 1.27 | 31 (1%)  3.78 / 3.51 / 1.32 |  |  |  |
| PLIC  Tstat / Pval / Dcoh | 174 (5%)  4.05 / 3.84 / 1.41 |  | 23 (1%)  3.74 / 3.47 / 1.30 |  |  |  |
| corona radiata |  |  |  |  |  |  |
| ACR  Tstat / Pval / Dcoh |  | 205 (3%)  3.68 / 3.39 / 1.28 | 428 (6%)  4.08 / 3.88 / 1.42 |  |  |  |
| SCR  Tstat / Pval / Dcoh | 2741 (29%)  4.21 / 4.05 / 1.47 |  | 2690 (27%)  4.36 / 4.23 / 1.52 |  |  |  |
| PCR  Tstat / Pval / Dcoh |  | 151 (8%)  3.65 / 3.35 / 1.27 | 183 (10%)  3.68 / 3.39 / 1.28 |  |  |  |
| PTR  Tstat / Pval / Dcoh |  | 92 (2%)  3.88 / 3.62 / 1.35 |  |  |  |  |
| general WM |  |  |  |  |  |  |
| Parietal WM  Tstat / Pval / Dcoh |  | 332 (3%)  3.70 / 3.42 / 1.29 |  |  |  |  |
| Frontal WM  Tstat / Pval / Dcoh | 144 (1%)  3.83 / 3.57 / 1.33 | 738 (3%)  3.91 / 3.67 / 1.36 |  |  |  |  |
