# Supplementary material for: Distinct Types of White Matter Changes Are Observed after Anterior Temporal Lobectomy in Epilepsy
Source: PLoS One. 2014 Aug 4;9(8):e104211. doi: 10.1371/journal.pone.0104211 (PMC4121328; doi:10.1371/journal.pone.0104211)
Supplement: File S1 — Joint histograms of the non-linear longitudinal registration of FA maps. (DOC) [file pone.0104211.s005.doc]

File S1

## Joint histograms of FA volumes before and after surgery coregistered with a non-linear algorithm in SPM. An approximately diagonal line is expected from well coregistered data.

## Green dots are calculated from whole-brain FA. Red dots show the histogram of the resection mask, which was excluded from the registration process. As one can see the green dots (the inclusive mask) consistently run along the diagonal reflecting good registration, whereas the red dots of the resection mask, excluded from the analysis, are largely off-diagonal.

|  |  |  |  |
| --- | --- | --- | --- |
|  |  |  |  |
|  |  |  |  |

|  |  |  |  |
| --- | --- | --- | --- |
|  |  |  |  |
|  |  |  |  |

|  |  |  |  |
| --- | --- | --- | --- |
|  |  |  |  |
|  |  |  |  |
